# Raoultella planticola Pneumonia in an Elderly Gardener: A Case Report and Literature Review

**DOI:** 10.7759/cureus.100402

**Published:** 2025-12-30

**Authors:** Sujeirys Paulino, Gilda Diaz-Fuentes

**Affiliations:** 1 Internal Medicine, BronxCare Health System, Bronx, USA; 2 Pulmonary and Critical Care Medicine, BronxCare Health System, Bronx, USA

**Keywords:** atypical pathogen, community-acquired infection, elderly patient, pneumonia, raoultella planticola

## Abstract

*Raoultella planticola* is a rare gram-negative bacillus, mostly found in the environment. It has been rarely identified as the source of infection, particularly in the respiratory tract, leading to shock and multiorgan failure. Limited cases have been reported in the past years, representing a diagnostic challenge.

We report a case of a 93-year-old African American female patient with a history of hypertension, type 2 diabetes, cerebrovascular disease, and dementia who presented with a two-week history of progressive respiratory symptoms, including dyspnea, productive cough, and altered mental status. She rapidly developed respiratory failure and shock, requiring intubation, mechanical ventilation, and vasopressor support. Bronchoalveolar lavage (BAL) cultures reported isolated pansensitive *R. planticola*. Further investigation revealed that the patient was an avid gardener with prolonged exposure to environmental soil and plant material, which is believed to be the source of the infection. She received treatment with intravenous ceftriaxone with the resolution of symptoms.

This case showcases the rare presentation of *R. planticola* pneumonia leading to shock and multiorgan failure in an elderly patient. It highlights the importance of considering atypical pathogens as a cause of community-acquired pneumonia, and the value of BAL in identifying rare organisms when initial cultures are negative and clinical deterioration persists.

## Introduction

*Raoultella planticola* is a gram-negative pathogen previously classified under the genus Klebsiella. However, it was noted that they belong to different species. This is important to note because misidentification can delay appropriate antimicrobial therapy and contribute to antibiotic resistance. *R. planticola* is commonly found in soil, plants, and aquatic environments, but human infections are rare [1-3].

Most reported cases have been described in immunocompromised or elderly individuals. Infection with this organism can range from urinary tract infection, pancreatitis, conjunctivitis, and bacteremia to pneumonia and respiratory failure [4-6].

This case is an example that patients with relative immunological impairment, such as those with diabetes mellitus, can also contract *R. planticola* infection, particularly following environmental exposure, resulting in severe disease, including bacteremia, pneumonia, acute renal damage, and respiratory failure needing mechanical support, which might arise from this infection spreading.

The case highlights the pathogenic potential of this organism and emphasizes the importance of clinical awareness.

## Case presentation

The patient is a 93-year-old African American woman with a medical history of hypertension, type 2 diabetes mellitus, cerebrovascular disease, and dementia who presented with a two-week history of shortness of breath, productive cough, malaise, decreased oral intake, and altered mental status. She had no toxic habits.

On arrival, she was found to be in respiratory failure, tachypneic and tachycardic with an oxygen saturation of 89% on ambient air, requiring administration of oxygen by nasal cannula.

Initial laboratory workup was positive for leukocytosis with white blood cell count (WBC) in 17.3 k/µL (reference 4.8-10.8), hypernatremia (152 mEq/L; reference 135-145), hyperkalemia (6.7 mEq/L; reference 3.5-5), and kidney injury with a BUN of 150 mg/dL (reference 8-26) and creatinine of 5.9 mg/dL (reference 0.5-1.5). Arterial blood gas revealed metabolic acidosis with pH 7.23, Bicarbonate 12 mEq/L, with increased lactate (2.7 mmol/L) (Table 1).

**Table 1:** Pertinent laboratory analysis on admission and before hospital discharge The discharge column highlights the improvement in inflammatory markers, acid-base disorders, electrolyte abnormalities, and kidney function following targeted antimicrobial therapy.

Parameters	On Admission	Discharge	Normal Range
White Blood Cell Count	17.3 k/ul	13.8 k/ul	4.8-10.8 k/ul
Neutrophils	90.3%	84%	40-70%
Sodium	152 mEq/L	135 mEq/L	135-145 mEq/L
Potassium	6.7 mEq/L	4.5 mEq/L	3.5-5 mEq/L
Bicarbonate (HCO₃⁻)	12 mEq/L	21 mEq/L	24-30 mEq/L
pH	7.23	7.42	7.35-7.45
Lactic Acid	2.7 mmol/L	1.4 mmol/L	0.5-1.6 mmol/L
Blood Urea Nitrogen (BUN)	150 mg/dL	46 mg/dL	8-26 mg/dL
Serum Creatinine	5.9 mg/dL	1.6 mg/dL	0.5-1.5 mg/dL

The patient was admitted to the intensive care unit and started on antibiotics, including vancomycin, ceftriaxone, and doxycycline. During the initial 24 hours of admission, her clinical condition worsened, and she developed respiratory failure that required intubation with mechanical ventilation, as well as septic shock requiring vasopressor administration.

**Figure 1:**
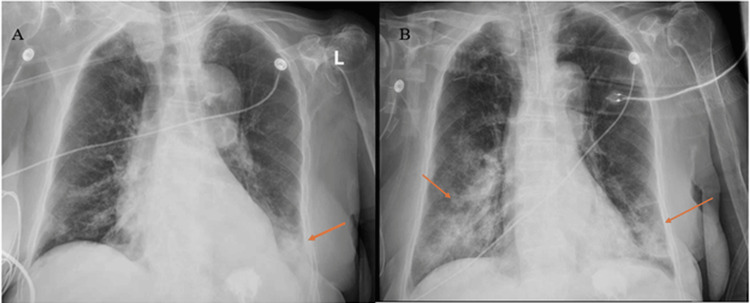
Initial X-rays of the chest CXR on admission showing left lower lobe infiltrate with small left pleural effusion (left) and CXR performed 24 hours after admission, with worsening bilateral pulmonary infiltrates (right).

Initial chest radiography (CXR) showed a left lower lobe infiltrate, which worsened over the subsequent 24 hours (Figure 1).

**Figure 2:**
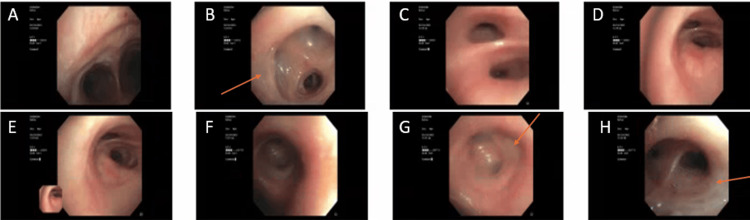
Fiberoptic bronchoscopy Tracheobronchial tree: (A) Carina, (B) right upper lobe with mucopurulent secretions (arrow), (C) bronchus intermedius, (D) right middle lobe, (E) right lower lobe, (F) left mainstem bronchus, (G) left upper lobe with mucopurulent secretions (arrow), (H) left lower lobe with mucoid secretions (arrow).

Respiratory viral panel, blood, urine, and respiratory cultures were negative. The patient underwent fiberoptic bronchoscopy with bronchoalveolar lavage (FOB-BAL) on the third day of admission, which revealed moderate bilateral mucopurulent secretions (Figure 2).

Respiratory cultures from FOB-BAL grew *R. planticola*, which was pan-sensitive to antibiotics, with the final culture report available after two days of specimen collection. Based on the sensitivity report, antibiotics were de-escalated to ceftriaxone, which was given for a total of 10 days.

**Figure 3:**
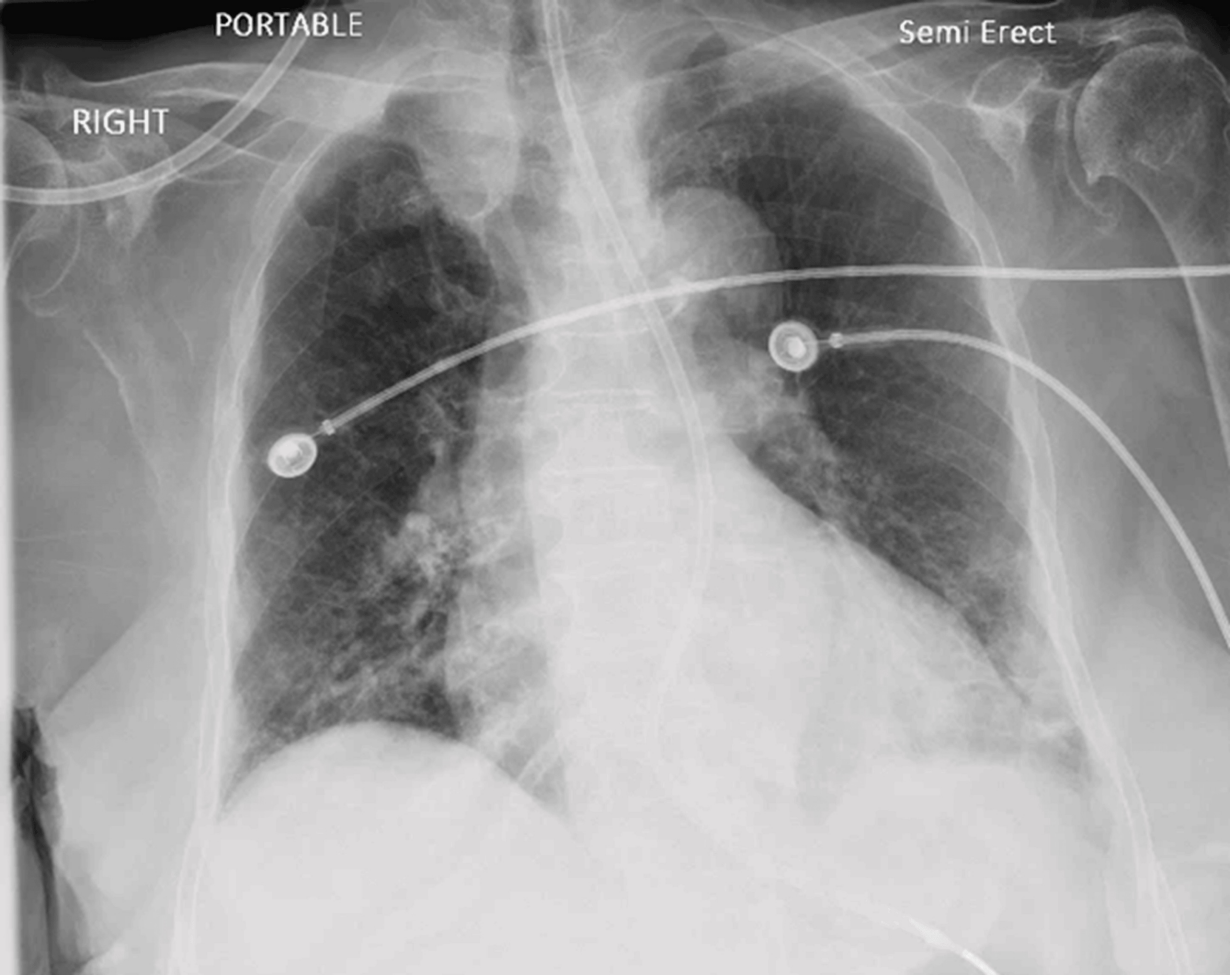
X-ray of the chest prior to discharge Chest x-ray performed before hospital discharge showed improvement in pulmonary infiltrates.

Later, the patient showed improvement in clinical status and laboratory derangements, including normalization of electrolytes and acid-base imbalances and kidney function, and improvement in X-ray findings (Figure 3).

The patient was an enthusiastic gardener who won community accolades for her horticultural contributions, which may have led to her exposure to *R. planticola* and subsequent infection, according to the patient's family, after additional discussion.

## Discussion

*Raoultella* species belong to the Enterobacteriaceae family of non-motile, encapsulated, Gram-negative rods. After phylogenetic analysis of 16S rRNA and rpoB gene sequences, *Raoultella*, which had previously been mistakenly categorized as Klebsiella, was eventually recognized as a distinct genus [1-3].

*R. planticola* is an environmental bacterium found in soil, water, wood, and plant material [1]. Traditionally considered harmless and non-pathogenic to humans. However, *R. planticola* has been implicated in a variety of infections, including conjunctivitis, liver abscess, cholangitis, bacteremia, pancreatitis, and urinary tract infections [4-7]. The upper respiratory and gastrointestinal tract have been reservoirs in humans, leading to respiratory, gastrointestinal, and systemic diseases [8]. Some studies report human colonization in 9% to 18% [3,9].

Most human infections have been reported in association with an immunocompromised state, especially malignancies, organ transplants, chronic pulmonary illness, invasive medical procedures, consumption of seafood, and exposure to soil, plants, or water contaminants [9-11].

In our patient, we believe she contracted the infection after exposure to soil during her gardening activities; it is unclear if the patient consistently used protective devices when gardening.

Gardening is a common hobby that is considered safe and relaxing; however, it is associated with a variety of infections, including those caused by soil, animals, or insect bites. The location of the garden and the characteristics of the soil are essential in determining the chances of infections. Infections associated with gardening are extensive, including fungal, parasitic, and bacterial infections [12].

Despite *R. planticola* being a common organism in the environment, human infections have been rarely reported. In addition, this organism is difficult to isolate in the laboratory and can be easily mistaken for Klebsiella species.

Pulmonary infections caused by this organism are rare, with only few cases reported. Most reported cases of *R. planticola* pneumonia occur in adults older than 40 years, particularly in the setting of immunosuppression or malignancy. Clinical manifestations vary from cough and dyspnea to a more severe presentation with fever, fatigue, altered mental status, and, in some cases, like ours, with respiratory failure and shock. In the Hong series, for instance, 11 patients developed pneumonia brought on by *R. planticola*, four patients (36.4%) required mechanical ventilation, and one patient (10%) passed away from shock and multiorgan failure [10]. The most frequent radiologic finding is unilateral or bilateral lung consolidation, followed by ground-glass opacification, pleural effusion, and micronodules [10,13,14].

The diagnosis of pneumonia is typically confirmed by respiratory culture, tracheal aspiration, or bronchoalveolar lavage.

Antibiotic treatment is based on the organism’s susceptibility; in general, *R. planticola* is generally sensitive to third-generation cephalosporins, carbapenems, fluoroquinolones, aminoglycosides, and beta-lactam/beta-lactamase inhibitors. However, resistance via extended-spectrum beta-lactamases and carbapenemases (e.g., blaIMP-8) has been documented [13-18].

The patient shared clinical features with previous reported cases of *R. planticola* pneumonia (Table 2), including advanced age and progression to respiratory failure and shock. However, unlike prior cases, this patient only had a mild immunocompromised state based on her age and previous diagnosis of type 2 diabetes, when compared to profound immunosuppression from malignancy, hematologic disorders, or organ transplantation, as described in the others.

**Table 2:** Reported cases of R. planticola pneumonia

Age & gender	Comorbidities	Clinical Presentation	Radiographic Findings	Diagnosis	Antibiotic Treatment	Outcome	Reference Number
77 Male	Non-small cell lung cancer	Respiratory failure (unclear oxygen requirement) shock	Right lower lobe bronchopneumonia	Sputum culture	Levofloxacin, Cefepime	Death	[7]
58 Male	Smoker, Heart Failure with reduced ejection fraction	Cough, dyspnea	Bilateral ground-glass opacities	Sputum culture	Piperacillin-Tazobactam +Levofloxacin> Piperacillin-Tazobactam	Recovery	[13]
36 Female	Smoker, Tracheal carcinoma	Cough, dyspnea	Right lower lobe consolidation	Sputum culture	Ceftriaxone > Piperacillin-Tazobactam	Recovery	[14]
41 Male	Smoker	Cough, dyspnea	Bibasilar nodules	Bronchoalveolar lavage	Benzylpenicillin and oral clarithromycin> Amoxicillin+Clarithromycin	Recovery	[15]
60 Male	Myeloid leukemia	Fever, dyspnea, respiratory failure requiring mechanical ventilation, shock,	Multifocal opacities	Sputum culture	Tigecycline, Levofloxacin	Death	[16]
71 Male	End-stage renal disease, Multiple myeloma, Hepatitis B	Cough, dyspnea, fever	Right lower lobe infiltrates	Sputum culture	Ceftriaxone> Levofloxacin	Recovery	[17]
43 Male	Metastatic pancreatic cancer	Fatigue, encephalopathy, hypoxic respiratory failure requiring high flow oxygen by nasal cannula	Left lower lobe infiltrates	Sputum culture	Ciprofloxacin, Vancomycin, and Piperacillin-tazobactam >Piperacillin-Tazobactam	Recovery	[18]
94 Female	CVA, Diabetes	Respiratory failure, shock, multiorgan failure	Bilateral consolidation	Bronchoalveolar lavage	Piperacillin and Tazobactam > Ceftriaxone	Survived	This case

Based on the gathered data, most patients develop mild to moderate symptoms, and severe presentations requiring mechanical ventilation and vasopressor support are uncommon, and at the same time, are associated with poor outcomes, especially in patients with cancer or hematologic disease.

In contrast, despite the development of respiratory failure requiring mechanical ventilation, septic shock, and acute kidney injury, our patient was able to achieve full recovery, implying that obtaining a positive clinical outcome may be significantly influenced by early detection and appropriate antibiotic treatment.

## Conclusions

*R. planticola*, although rare, should be considered a potential pathogen in respiratory infections. The elderly and immunocompromised patients are the most commonly affected. Early recognition and prompt antibiotic treatment are key to achieving good outcomes. This case also emphasized that, when dealing with rare organisms, a detailed clinical history, including potential environmental exposures, can be important for reaching the correct diagnosis.
